# A tale of flawed e-cigarette research undetected by defective peer review process

**DOI:** 10.1007/s11739-022-03163-x

**Published:** 2022-12-08

**Authors:** Riccardo Polosa, Konstantinos Farsalinos

**Affiliations:** 1 Center of Excellence for the Acceleration of Harm Reduction (CoEHAR), University of Catania, Catania, Italy; 2 Department of Clinical and Experimental Medicine, University of Catania, Catania, Italy; 3 Institute of Internal Medicine, AOU “Policlinico–V. Emanuele”, Via S. Sofia, 78, Catania, Italy; 4 ECLAT Srl, Spin-off of the University of Catania, Catania, Italy; 5 Smoking Behavior Research Unit, Department of Public and Community Health, University of West Attica, Athens, Greece

## Commentary

Research on tobacco harm reduction (THR), and particularly on electronic cigarettes (e-cigarettes), remains a highly controversial topic in the scientific community. The controversy is also sustained by research, which is often poorly designed, conducted, and interpreted [1]. Dissemination of inaccurate information on smoke-free alternatives in the media contributes to public skepticism and uncertainty, particularly among smokers, who as a result are discouraged from adopting reduced-risk lifestyles.

Due to the limited evidence on the health impact of e-cigarettes from longitudinal cohorts, several cross-sectional studies have been published instead, mostly showing that e-cigarette use may be associated with diseases of the respiratory and the cardiovascular system. For example, Parekh et al. analyzed the 2016 and 2017 Behavioral Risk Factor Surveillance System (BRFSS), a cross-sectional population survey in the United States, to examine the “risk of stroke” with e-cigarette use [2]. They reported that switching from combustible cigarettes to e-cigarettes does not confer stroke benefits, but e-cigarette users who were former or current cigarette smokers had significantly higher odds of stroke even compared with current smokers. Discussion about “risk” of stroke clearly implies a specific temporal sequence of events, i.e., that exposure to the “risk factor” (in this case, to e-cigarettes) precedes the development of the disease (stroke) [3]. However, no information on the timing of e-cigarette use initiation and of stroke occurrence was available in BRFSS.

This is not an isolated case. Cross-sectional data such as BRFSS and National Health Interview Survey (NHIS) do not contain any information on exposure initiation or diagnosis. Therefore, they should not be used to make causal inferences, unless questions that would generate information about the age of disease diagnosis and of tobacco and nicotine use initiation were introduced. Despite these unacknowledged limitations, BRFSS and NHIS data have been used to show relationships between e-cigarette use and smoking-related diseases in multiple papers, which have been often accompanied by press statements communicating messages that could be interpreted as causal inferences. Consequently, these studies on e-cigarettes are reiterating the same potential mistake confusing associations with causation, leading to unreliable conclusions [1]. Remarkably, although specific information about age of e-cigarette initiation and timing of first diagnosis are available in the Population Assessment of Tobacco and Health Survey (PATH), these have been overlooked.

To examine the reliability of associations found in cross-sectional studies, Rodu and Plurphanswat have used data from the PATH Wave 1, which has information about the age of disease diagnosis and tobacco and nicotine use initiation [4].

The authors have provided cogent and convincing evidence based on a creatively simple assessment of data from the first wave of the PATH survey that smoking-related diseases (COPD, emphysema, myocardial infarction and stroke) were only rarely diagnosed in respondents who had initiated e-cigarette use prior to the age of diagnosis of these disorders, while, in marked contrast, these diseases were nearly always diagnosed following (mostly many years after) the age of initiation of smoking. Disease cases among smokers that definitely occurred after first exposure represented 97% of all cases for COPD, 96% for emphysema, 98% for myocardial infarction and 93% for stroke. Indeed, most of these diseases were ultimately diagnosed in respondents who initiated smoking prior to 18 years of age. As the authors correctly point out, cross-sectional population-based data that fail to include data on the age of initiation of e-cigarette and combustible cigarette use cannot be relied on for drawing conclusions regarding potentially causal associations with typical smoking-related diseases. This is further complicated when you take into account the duration of exposure and the fact that most adult e-cigarette users are current or former smokers.

What the authors accomplished was so conceptually simple and fundamentally important that we are very surprised that no one, particularly any of the authors of the PATH survey publications, had carried out a similar assessment previously. Moreover, reviewers of the many PATH, BRFSS, and NHIS based papers showing a relationship between e-cigarette use and smoking-related diseases appear not to have considered the critical importance of the timing of events.

And this relates to the next question. How was it possible that the peer review process at highly respected scientific Journals has failed to detect such fatal flaws and has allowed publication of low-quality papers lacking inclusion of such key factors essential for the interpretation of their analysis? The unopposed acceptance of these (low-quality) papers by prestigious journals is symptomatic of a significant dysfunction in scientific publishing, which is distorting the practice of science. Of course a problem with peer review is that it often does not detect errors, including some very major mistakes [5]. But this is understandable given the well-known limitations of the peer review process. However, the main problem is that in the context of highly polarized scientific debates (as in e-cigarette research) the peer review process becomes strongly biased for or against a certain narrative. Peer review might be described as a process where the 'establishment' decides what is important and what is not. There are many examples of peer review turning down hugely important work or praising scientific lies [6].

The findings by Rodu and Plurphanswat are an important reminder that association should not always be interpreted as causation [4]. This is crucial not only for study manuscripts, which usually exemplify this limitation, but also for relevant press releases which often make oversimplified causal interpretations that may be highly misleading. Additionally, the authors showed the possibility for reverse causation, i.e., that having a diagnosis for respiratory or cardiovascular disease might be the reason for smokers to switch to e-cigarette use [4]. Finally, they provide useful insight for improving the structure of cross-sectional survey questionnaires. Inclusion of questions about the timing of disease diagnosis and of initiation of exposure to smoking or alternative nicotine products appears to be an appropriate measure to reduce the risk of misinterpreting associations and to more accurately explore the link between nicotine products and disease development.
