# Correction to: A tale of flawed e‑cigarette research undetected by defective peer review process

**DOI:** 10.1007/s11739-022-03183-7

**Published:** 2022-12-27

**Authors:** Riccardo Polosa, Konstantinos Farsalinos

**Affiliations:** 1 Center of Excellence for the Acceleration of Harm Reduction (CoEHAR), University of Catania, Catania, Italy; 2 Department of Clinical and Experimental Medicine, University of Catania, Catania, Italy; 3 Institute of Internal Medicine, AOU “Policlinico–V. Emanuele”, Via S. Sofia, 78, Catania, Italy; 4 ECLAT Srl, Spin-off of the University of Catania, Catania, Italy; 5 Smoking Behavior Research Unit, Department of Public and Community Health, University of West Attica, Athens, Greece

**Correction to: Internal and Emergency Medicine** 10.1007/s11739-022-03163-x

In this article the authors name Riccardo Polosa, Konstantinos Farsalinos was incorrectly written as Polosa Riccardo, Farsalinos Konstantinos.

The original article has been corrected.

